# The Veterinary Medical Society of the State of New Jersey and Vicinity

**Published:** 1897-06

**Authors:** E. Buckley

**Affiliations:** Secretary


Secretary.

### THE VETERINARY MEDICAL SOCIETY OF THE STATE OF NEW JERSEY AND VICINITY.

THE annual meeting was held at Dr. L. R. Sattler's residence in Newark, N. J., on April 13th. The following officers were elected for the ensuing year: Dr. L. R. Sattler, President; Dr. E. L. Loblein, Vice-President; Dr. E. R. Ogden, Treasurer; Dr. E. Buckley, Secretary. The Society elected a number of new members, which will give it a new impetus.

Papers were prepared, but the time being consumed by the election of officers, they were postponed until the next meeting. A number of cases were cited and freely discussed by the members, namely, purpura, parturient apoplexy, and thrombosis caused by abscess in the lung.

THE regular monthly meeting was held at Dr. Sattler's hospital, in Newark, on Tuesday, May 11th. Called to order at 4.30 P.M., President Sattler in the chair. The following members responded to roll-call: Drs. Sattler, Turner, Ogden, Loblein, Zucker, and Buckley. The minutes of the last meeting were read and approved.

Dr. Knott, of Plainfield, was elected a member. Dr. Sattler made a few suitable remarks regarding the welfare of our Society.

Discussions and reports from practice were next in order. Dr. Sattler reported having treated a case of chronic rheumatic shoulder-lameness with subcutaneous injections of veratrine in alcoholic solution in two-decigramme doses. Two injections were made around painful tissues. The injections were followed by very large abscesses. The animal made a good recovery. Discussion followed as to whether any other counter-irritant would not have produced the same beneficial results. The nature and formation of schirrous cords were freely discussed; also vaginal tumors in bitches.

Dr. Turner, of Hackensack, promised to prepare a paper to be read at the next meeting.

E. BUCKLEY, V.S.,
Secretary.

